# Relationship between spontaneous EEG oscillations at 7 and 45 days of acute plateau exposure and the plateau acclimatization index

**DOI:** 10.3389/fnins.2026.1830546

**Published:** 2026-06-18

**Authors:** Qingqing Liu, Peng Dang, Hao Li, Niannian Wang

**Affiliations:** Xizang Autonomous Region Key Laboratory for High Altitude Brain Science and Environmental Acclimatization, Xizang University, Lhasa, China

**Keywords:** hypoxia, inter-regional coupling, multi-scale sample entropy, plateau adaptation habituation index, resting-EEG

## Abstract

**Introduction:**

This study tracked 47 high-altitude migrants to investigate the adaptive mechanisms of the brain to hypoxic environments.

**Methods:**

EEG and physiological indicators (SpO_2_, HCT, AAI) were recorded during the acute (7 days) and chronic (45 days) phases of high-altitude exposure. EEG complexity was assessed using multiscale entropy (MSE), and inter-regional brain coupling was also analyzed.

**Results:**

Compared with the chronic phase, EEG complexity in the frontal, parietal, and occipital lobes was higher in the acute phase, whereas inter-regional brain coupling was stronger in the chronic phase. SpO_2_ decreased during the acute phase and then slowly recovered; HCT continued to rise; AAI showed a decelerating downward trend. Correlation analysis revealed that SpO_2_ was negatively correlated with fine-scale MSE, and HCT was negatively correlated with medium- to coarse-scale MSE. AAI was correlated only with occipital MSE during the acute phase. During the chronic phase, AAI was negatively correlated with MSE coupling across multiple brain regions but not with MSE itself.

**Discussion:**

These findings suggest that hypoxia may increase fine-scale complexity by enhancing local neural interconnections, whereas elevated HCT reduces long-range interactions between distributed neural populations. The brain exhibits a compensatory pattern of “complexity reduction with enhanced inter-regional coupling” during hypoxic adaptation, which may represent an optimization of neural efficiency under sustained hypoxia.

## 1 Introduction

The plateau environment, as a special geographical region with an altitude of more than 2,500 m (Shen et al., 2024), is typified by the Tibetan Plateau (with an average altitude of more than 4,000 m), whose low oxygen partial pressure, strong ultraviolet radiation and extreme climatic characteristics constitute a unique biological research scenario. With the continuous expansion of the plateau active population, the adaptation mechanism of the organism to the hypoxic environment has become an interdisciplinary research hotspot, in which the dynamic adjustment of the brain’s cognitive function in extreme environments has attracted particular attention.

Existing research suggests that hypoxic exposure leads to complex cognitive impairment. The acute phase (within 7 days of Tibet entry) is characterized by blunted visual and auditory perception and weakened executive control (Zhu and Fan, 2017); long-term exposure (more than 1 month) further involves working memory and psychomotor abilities (Chen et al., 2017). However, there is significant heterogeneity in findings – some studies have shown that cognitive performance in plateau-dwelling adolescents is not limited by hypoxia (Yu et al., 2022), and simulated chamber experiments have confirmed that acute hypoxia (1 h) does not alter language processing abilities (De Bels et al., 2019). This contradiction highlights the critical influence of individual differences, exposure environment (field vs. simulation), testing instruments and sample characteristics on study results, and the urgent need to establish a unified analytical framework through the integration of emerging neurocognitive technologies.

Traditional physiological monitoring indicators are valuable in the assessment of hypoxia. It has been found that blood oxygen saturation (SpO₂) decreases non-linearly with altitude (Woorons and Richalet, 2021), and erythrocyte pressure volume (HCT) shows a compensatory increase (Richardson et al., 2008). However, SpO₂ is susceptible to peripheral circulatory disturbances (Xu et al., 2006), and there are non-hypoxia-related fluctuations in HCT (Zhou and Pang, 2023), which prompted researchers to propose a composite index, the Altitude Acclimation Acclimatisation Index (AAI=SpO₂/HCT), with a critical value of 1.7228 that effectively distinguishes acclimatization status (Li et al., 2024) effectively differentiate the acclimatization status (Li et al., 2024). In this study, we will combine SpO₂, HCT and AAI to construct a multidimensional physiological assessment system.

Neurophysiological techniques provide a noninvasive window for revealing functional changes in the hypoxic brain. Electroencephalography (EEG) is an excellent neurophysiological method for studying hypoxia due to its high temporal accuracy, relatively low cost, and portability (Hutcheon et al., 2023). Many studies have already employed EEG to investigate changes in brain function under hypoxic environments. For example, a cross-sectional study of soldiers stationed at different altitudes found that as altitude increased, the amplitude of event-related potential components related to response inhibition (e.g., NoGo-N2) significantly decreased and was positively correlated with blood oxygen saturation (SpO_2_), suggesting that hypoxic exposure impairs executive function. Furthermore, from the perspective of resting-state functional connectivity, this study compared migrants with different durations of high-altitude residence (1–2 years, 8–10 years, 18–20 years) and found that with increasing residence duration, functional connectivity strength decreased in the frontal lobe but increased in the occipital lobe, indicating that the brain may compensate for frontal lobe impairment by enhancing occipital lobe function, thereby better adapting to high-altitude hypoxic environments (Wei et al., 2021). Another time-domain and frequency-domain EEG study under high-altitude conditions revealed that within the 200–800 ms time window, the high-altitude group exhibited higher amplitudes and broader activation ranges in the occipital lobe compared to the low-altitude group. Frequency-domain analysis showed that the low-altitude group displayed dispersed alpha-band activation in the frontal and central lobes, whereas the high-altitude group exhibited concentrated beta-band activation, with both groups showing stronger activation in the frontal and central lobes and weaker activation in the occipital lobe (Ji et al., 2024). These findings collectively suggest that high-altitude environments induce spatiotemporal reorganization of EEG activity, which may reflect adaptive regulatory mechanisms of the brain under hypoxic conditions.

Resting-state EEG being widely used in EEG studies due to its ease of use and cost-effectiveness (Zebhauser et al., 2023), it is widely used in EEG research. Compared with the task state, resting-state EEG activity is more objective and generally has better retest reliability than the task state, of which the closed-eye resting state has higher retest reliability than the open-eye resting state (Qin et al., 2023), which can effectively avoid data quality problems due to the practice effect and so on, and therefore, closed-eye resting-state EEG data will be collected in the present study for investigation. Multiscale sample entropy (MSE) is able to capture the dynamic adaptation process of the brain to hypoxia by quantifying the change in complexity of EEG signals over different time scales (Gu et al., 2022). In this study, we will focus on the evolution of EEG characteristics from the acute acclimatization period (at 1 week of exposure) to the chronic acclimatization period (at 6 weeks of exposure), with an emphasis on analyzing the correlation between MSE and physiological indices, to reveal the mechanism of hypoxia-induced reorganization of brain function.

Based on previous findings: the decline in cognitive function is most pronounced during the acute acclimatization period (1 week), and as the duration of exposure to high altitude increases, the body gradually achieves habituation, resulting in partial recovery of cognitive function (Zhu and Fan, 2017; Jiang et al., 2011). This study hypothesizes that: elevated EEG complexity in the acute phase reflects compensatory neural resource recruitment, and enhanced functional connectivity in the chronic phase reflects network optimization; AAI is able to more sensitively track the trajectory of cognitive function recovery by integrating oxygenation efficiency and blood oxygen carrying capacity. Through the longitudinal tracking design, this study will provide multidimensional evidence for the neurocognitive adaptation mechanism of plateau hypoxia and promote the development of brain health protection strategies for people working in extreme environments.

## 2 Materials and methods

### 2.1 Participants and procedure

We recruited 61 Han Chinese university students from the central plains of China (less than 1,000 m above sea level) who were admitted to Tibet University. These participants had never lived at high altitude before. We recruited the participants through an online platform before the start of the academic year at Tibet University, and they were asked to go to the hospital to have their SpO₂ and HCT measured 3 days before entering high altitude. SpO₂, HCT, and resting EEG data were collected at 7 and 45 days after the participants entered the altitude. Participants participated in the tests at 9 a.m., 12:30 p.m., or 3:30 p.m. First, the SpO₂ monitor was mounted on the index finger of the left hand (yuwell YX306 pulse oximeter), and then resting EEG data were collected with eyes closed for 5 min. Of these, 6 withdrew from the experiment midway; 8 were excluded due to excessive eye movement artifacts, head movements and muscle artifacts with >50% contamination during acute or chronic hypoxia. Among the eight excluded participants, six exhibited artifacts predominantly during the acute adaptation period, manifesting as increased electromyographic activity or ocular/movement artifacts. In contrast, the proportion of artifacts decreased during the chronic adaptation period. The remaining two participants showed consistently high artifact rates across both phases. This suggests that physiological discomfort during the early stage of acute hypoxic exposure (e.g., tension, altered respiratory rhythm, or poor sleep) may have induced more involuntary movements or electromyographic activity in the participants, thereby affecting EEG data quality. After these exclusions, we statistically analyzed the data of the remaining 47 participants. These participants (males: 22; females: 25) had a mean body mass index of (21.34 ± 0.41) (range: 17.04–32.19). All participants were in good health and free of neurological or psychiatric disorders, brain injury, or drug use. All participants gave written informed consent and paid the participation fee. The study was approved by the local ethics committee of Tibet University and followed the Declaration of Helsinki.

### 2.2 EEG recording and preprocessing

The EEG data were acquired utilizing the ANT Neuro 64-electrode system^1^, with a sampling frequency of 1,000 Hz. Electrode placement adhered to the standard 10–20 system, maintaining all impedances below 5 kΩ. The online reference electrode was placed on CPz, and the ground electrode was placed on FCz. The amplifier enhanced the signal to facilitate continuous EEG recording. Online filtering was executed with a bandwidth ranging from 0.01 to 100 Hz, during a resting state with eyes closed for a 5-min duration. Data processing and analyses were performed using the MATLAB toolbox EEGLAB (Delorme and Makeig, 2004) and Fieldtrip (Oostenveld et al., 2011) in MATLAB (version 2021b, The MathWorks). Raw data were downsampled to one-fourth (250 Hz) of the original sample rate before processing.

For offline processing of EEG data, EEGLAB (Delorme and Makeig, 2004), an open-source toolbox within the MATLAB environment (version 2021b, The MathWorks, Inc., Natick, MA) was employed. The continuous EEG data underwent high-pass filtering at 2 Hz, with data below 20 Hz receiving low-pass filtering through a rudimentary FIR filter. Identification of potentially bad electrodes was followed by data correction via spherical interpolation. Subsequently, successive 0.5-s periods were extracted, with contaminated periods being manually marked and discarded.

The Independent Component Analysis (ICA) algorithm (Jung et al., 2001) was utilized for the rectification of EEG data contaminated by muscle, heart, line noise, and channel noise. Employing the ICLabel plug-in (Pion-Tonachini et al., 2019), components showcasing a probability exceeding 0.6 for electromyography, electrocardiogram, line noise, and channel noise were removed (average removal entailed 5 components per subject, SD = 2.0). Epochs manifesting amplitudes beyond ±100μv were excluded. Lastly, the EEG data were re-referenced to the average reference, culminating the data processing sequence. Forty-seven participants’ at the minimum data size of 180 s(s). To minimize the potential impact of varying data lengths between participants on further analyses, all participants’ data were cropped to the first 180 s.

### 2.3 Brain signal complexity: multiscale entropy (MSE) analysis

We investigated the complexity of brain signals across various temporal scales using the Multiscale Entropy (MSE) analysis method. MSE is a powerful tool that quantifies the complexity of a time series by assessing its variability and predictability at different scales, providing insights into the information processing capabilities of neuronal systems. Specific EEG channels and epochs were selected for analysis based on the experimental design and task requirements. Sample entropy, a fundamental component of MSE, was calculated with an embedding dimension (m) of 2 and a tolerance level (r) of 0.2 times the standard deviation of the time series, which are commonly used parameters in EEG analysis. MSE was computed by first constructing coarse-grained time series at different time scales, where at time scale 1, the coarse-grained time series was the original signal itself, and for time scale n, it was formed by averaging n consecutive time points from the original signal. Sample entropy was then calculated for each coarse-grained time series using the method proposed by Richman and Moorman (2000). MSE was computed across a range of time scales, typically from 1 to 15, to capture the complexity of the signal at different temporal resolutions, and the resulting MSE values were analyzed to assess the changes in signal complexity across time scales, providing insights into the underlying neural dynamics. To facilitate analysis, time scales were categorized into three groups: fine (1–5), medium (6–10), and coarse (11–15), based on previous studies that have shown distinct neural processes operating at these scales. Average sample entropy was calculated for each participant and condition to assess differences in brain signal complexity at different levels of altitude exposure, and statistical analysis was performed. To investigate the association between physiological changes and brain signal complexity under different altitude exposure conditions, we calculated the Pearson correlation coefficient between average sample entropy and physiological indicators (SpO₂, AAI, and HCT) at each time scale. By employing these rigorous methods, this study aims to comprehensively assess the complexity of brain signals at different time scales through MSE analysis and explore their relationship to physiological changes under different altitude exposure conditions.

Based on the brain region segmentation method described by Chen et al. (2022), we conducted a comprehensive analysis of the multi-scale sample entropy for each subject. This analysis covered the entire brain (including all channels) and specifically focused on six regions of interest (ROI): (i) the frontal lobe (FP2, Fz, FP1, F3, F7, F8, F4), (ii) the frontal central region (Cz, FC1, C3, C4, FC2), (iii) the parietal lobe (CP1, P3, Pz, CP2, P4), (iv) the left temporo-parietal junction (FC5, FT9, T7, CP5, P7), (v) the right temporo-parietal junction (P8, CP6, T8, FT10, FC6), and (vi) the occipital lobe (O1, Oz, O2). The specific electrode layout and ROI divisions are illustrated in Supplementary Figure 1.

### 2.4 Statistical analyses

Changes in physiological levels during different periods of hypoxia exposure: A linear mixed-effects model (Bates et al., 2015) was used to explore changes in physiological indices during different periods of hypoxia exposure. To test whether there were differences in SpO₂/HCT/AAI during different periods of hypoxia, we used hypoxic conditions (acute vs. chronic hypoxia) as a fixed effect, subjects as a random effect, and SpO₂/HCT/AAI as dependent variables, respectively.

Differences in MSE across exposure periods (within-subjects study): for each region of interest (ROI), the signals were averaged across all channels within the ROI to obtain a representative time series. We used paired *t*-tests to explore within-subjects changes in MSE across exposure periods. Specifically, we compared MSE on three scales for six regions of interest (ROIs) during the acute exposure period (7 days) and the chronic exposure period (45 days).

Differences in coupling between brain regions during different exposure periods: to explore differences in coupling between brain regions during different exposure periods, we calculated the sample entropy coupling (i.e., correlation) between all six pairs of ROIs. We then compared the coupling between acute and chronic exposure periods using paired *t*-tests.

Associations between physiological indices during different exposure periods and MSE at each time scale: we performed partial correlation analyses to explore associations between physiological indices (SpO₂, erythrocyte compression (HCT), AAI) and MSE at different time scales during the acute and chronic exposure periods. Covariates: AAI/SpO₂/HCT, sex, BMI at baseline. We performed analyses separately for each exposure period to assess associations between physiological changes and brain signaling complexity.

Associations between physiological indices and inter-cerebral coupling during different exposure periods: to investigate the relationship between physiological indices and coupling between brain regions, we performed partial correlation analyses between SpO₂, HCT, AAI and inter-cerebral coupling during the acute exposure period and the chronic exposure period, respectively. Covariates: AAI/SpO₂/HCT, sex, BMI at baseline: we analyzed each exposure period separately to assess the association between physiological changes and coupling between brain regions.

## 3 Results

### 3.1 Changes in physiological levels during different periods of hypoxic exposure

**Figure 1:**
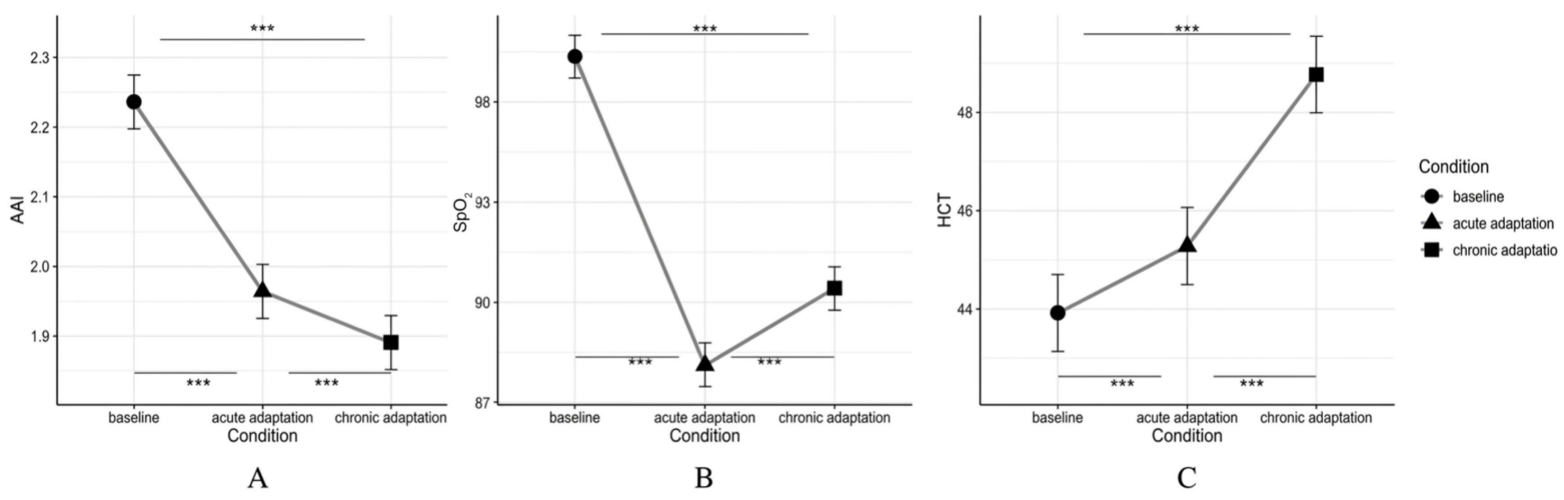
Differences in changes in AAI, SpO₂, and HCT across adaptation periods. **(A)** AAI, **(B)** SpO₂, **(C)** HCT. All post hoc combinations of ANOVA comparisons are significant at *p* < 0.001. Vertical bars in the graph indicate 95% confidence intervals for the repeated measures mean difference estimates. Baseline represents the plains period.

The results of the linear mixed-effects model showed a significant decrease in AAI levels during both the acute acclimatization period (*t* = −10.64, *p* < 0.001) and the chronic acclimatization period (*t* = −15.14, *p* < 0.001), and a significant decrease in AAI levels during the chronic acclimatization period relative to the acute acclimatization period (*t* = −3.19, *p* < 0.001), relative to the low altitude period (see Figure 1A). SpO₂ levels were significantly lower during both the acute acclimatization period (*t* = −19.03, *p* < 0.001) and the chronic acclimatization period (*t* = −17.10, *p* < 0.001) compared to the low altitude period and, furthermore, SpO₂ levels during the chronic acclimatization period were significantly higher (*t* = 4.73, *p* < 0.001) than during the acute acclimatization period (see Figure 1B). HCT levels were significantly higher in both the acute acclimatization period (*t* = 2.84, *p* = 0.006) and chronic acclimatization period (*t* = 10.42, *p* < 0.001) compared to the low altitude period and were significantly higher in the chronic acclimatization period than in the acute acclimatization period (*t* = 9.48, *p* < 0.001) (see Figure 1C).

### 3.2 Differences in migrants’ MSE across exposure periods

**Figure 2:**
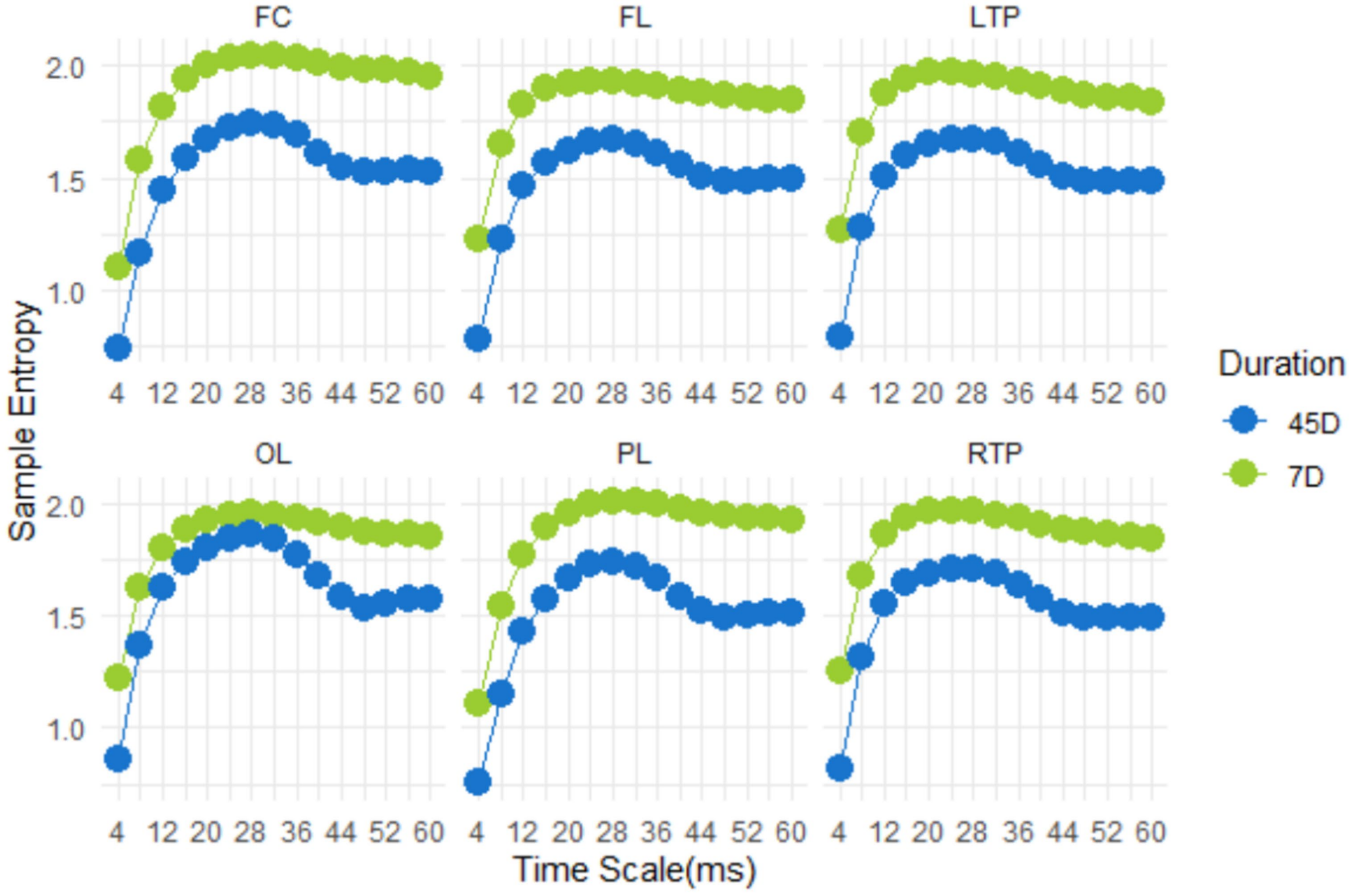
Multiscale entropy for the acute exposure period (7 days) versus the chronic exposure period (45 days). Across the six brain regions, the acute period exhibited greater sample entropy than the chronic period, both on the coarse and fine time scales. Time scales 1–15 were converted to milliseconds by dividing each time scale by the sampling rate (250 Hz) and multiplying the resulting number by 1,000 milliseconds.

Figure 2 demonstrates the differences in MSE of the 6 ROI brain regions in migrants at different exposure periods. In order to deeply explore the changes of MSE on different SCALEs in migrants during different exposure periods, we used paired *t*-tests to examine the MSE of the 6 ROIs at 3 SCALEs in different exposure periods, respectively. The results showed that the MSE of all 6 brain regions at different SCALEs was significantly higher at the acute exposure period (7 days) than at the chronic exposure period (45 days) (*p* < 0.001, except for the OL brain region at SCALE 6–10, *p* = 0.013). Therefore, it can be concluded that at the acute exposure period (7 days), migrants showed greater brain signal complexity in any brain region compared to the chronic exposure period (45 days).

### 3.3 Differences in coupling between brain regions during different exposure periods

**Figure 3:**
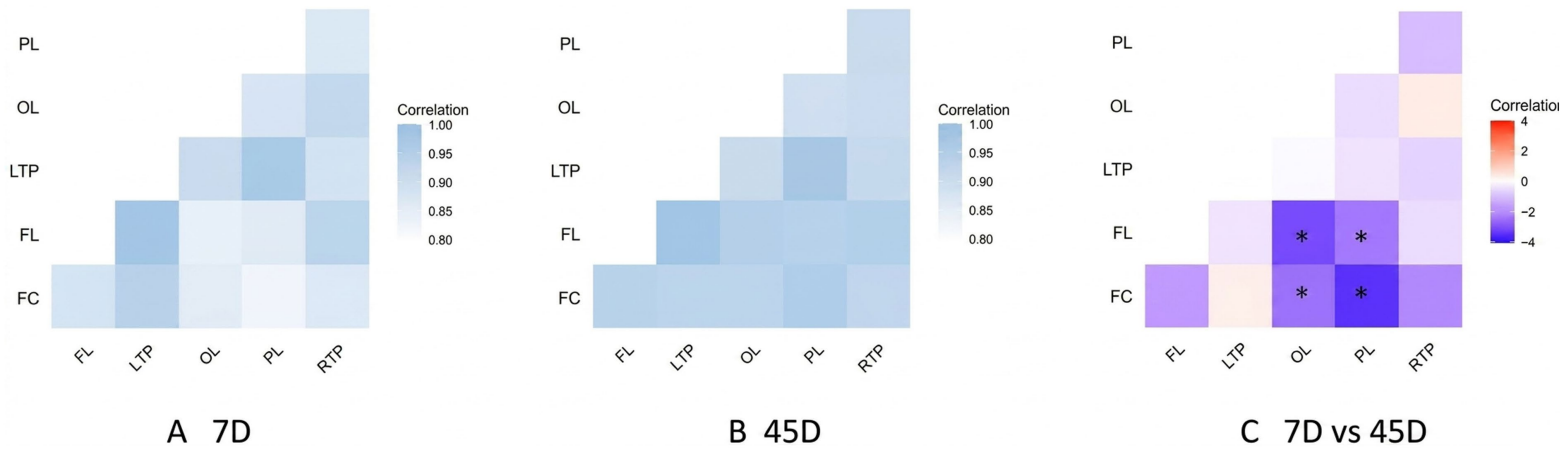
**(A)** MSE coupling between brain regions at the time of acute phase (7D). **(B)** MSE coupling between brain regions at the time of chronic phase (45D). **(C)** MSE coupling between brain regions comparing the acute and chronic phases. The results showed that MSE coupling between brain regions (FL-PL, FL-OL, FC-PL, and FC-OL) was significantly higher during the chronic exposure period (45D) than during the acute exposure period (7D). *Represents *p* < 0.05.

**Figure 4:**
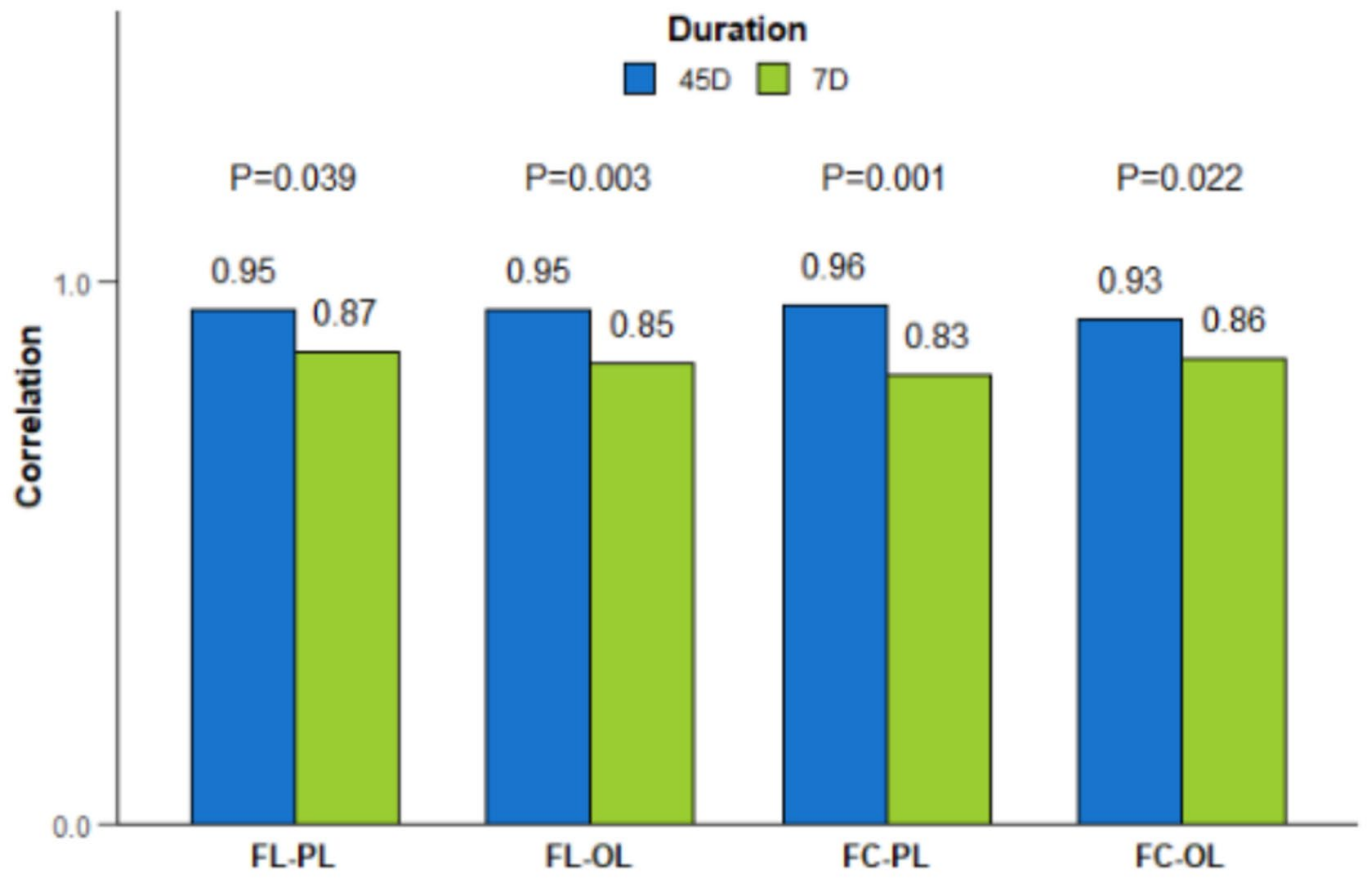
Differential analysis of MSE coupling between brain regions (FL-PL, FL-OL, FC-PL, FC-OL) comparing the acute phase to the chronic phase.

To explore differences in the coupling between brain regions across exposure times, we calculated the pairwise correlations of sample entropy between the six brain regions. These relationships are shown in Figure 3. The results showed that the MSE coupling between brain regions (FL-PL, FL-OL, FC-PL, and FC-OL) was significantly higher during the chronic exposure period (45 days) than during the acute exposure period (7 days) (see Figures 3, 4). This suggests that the inter-regional coupling between FL-PL, FL-OL, FC-PL, FC-OL brain regions was stronger in migrants during the chronic exposure period compared to the acute exposure period.

### 3.4 Associations between physiological indicators and MSE across time scales for different exposure periods

**Figure 5:**
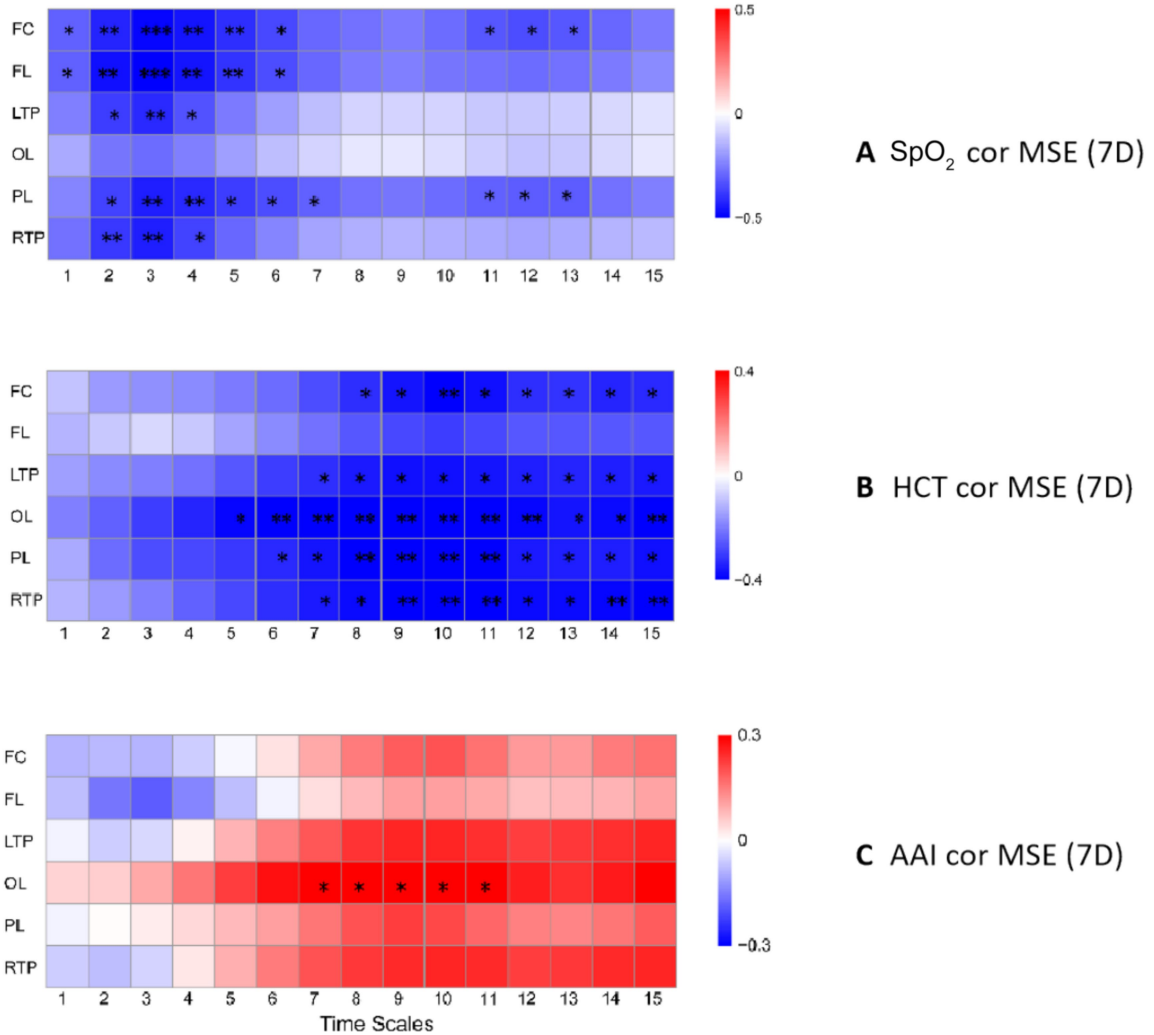
Correlation analysis of AAI with each of the 6 brain regions on 15 time scales (covariates: AAI, gender, BMI at baseline) during the acute exposure period (7 days). **(A)** SpO₂ cor MSE (7D), **(B)** HCT cor MSE (7D), **(C)** AAI cor MSE (7D). Among them, AAI was positively correlated with MSE in OL brain regions at 7–11 time scales. *Represents *p* < 0.05.**Represents *p* < 0.01; ***Represents *p* < 0.001.

**Figure 6:**
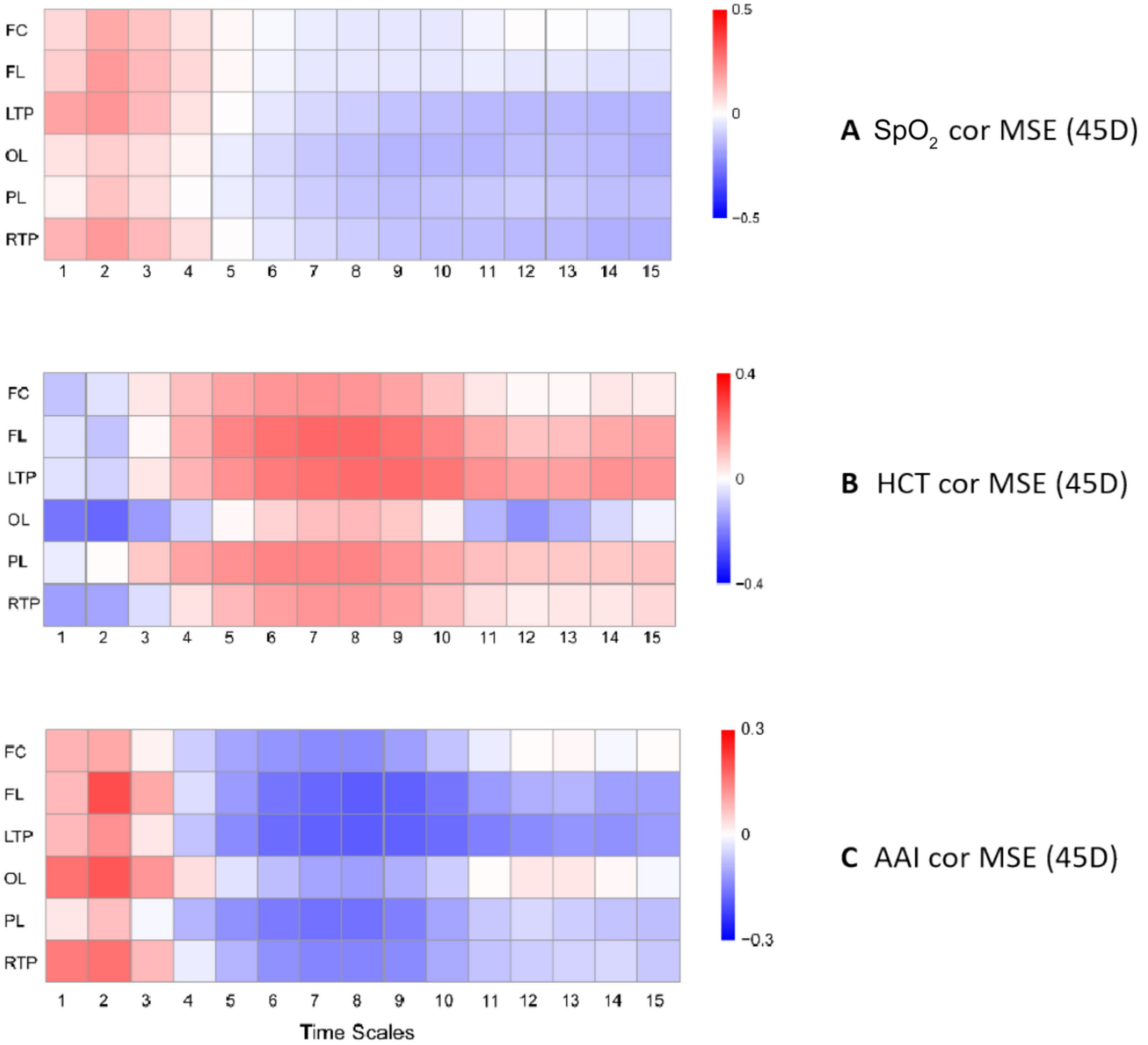
Bias correlation analysis during the chronic exposure period (45 days). **(A)** Correlation between SpO₂ and MSE across 6 brain regions on 15 time scales; **(B)** Correlation between HCT and MSE; **(C)** Correlation between AAI and MSE. Covariates include AAI, gender, and BMI at baseline.

In order to explore the association between changes in physiological indicators and brain signal complexity, we analyzed the correlations between SpO₂, HCT, AAI and MSE during the acute and chronic exposure periods, respectively. It was found that during the acute exposure period, SpO₂ was significantly and negatively correlated with MSE at finer granularity (SCALE range 1–6), HCT was significantly and negatively correlated with MSE at coarser granularity (SCALE range 6–15), and AAI was significantly and positively correlated with MSE at intermediate granularity (SCALE range 7–11) in the OL brain region (see Figure 5). However, during the chronic exposure period (45 days), we did not observe a direct significant correlation between SpO₂, HCT, AAI and MSE (see Figure 6). Further observation revealed that both SpO₂ and HCT were lower in the acute phase compared to the chronic phase, suggesting that relatively lower SpO₂ and HCT in the acute exposure phase were associated with relatively higher MSE. Meanwhile, AAI was higher in the acute phase than in the chronic phase, and AAI was significantly and positively correlated with the MSE of the intermediate granularity in the OL brain region, revealing that the higher MSE of the OL brain region in the acute phase was associated with AAI. This shows that the relatively higher MSE in each brain region at three time scales during the acute exposure period was closely related to the changes in SpO₂, HCT, and AAI.

### 3.5 Associations between physiological indicators and coupling between brain compartments during different exposure periods

**Figure 7:**
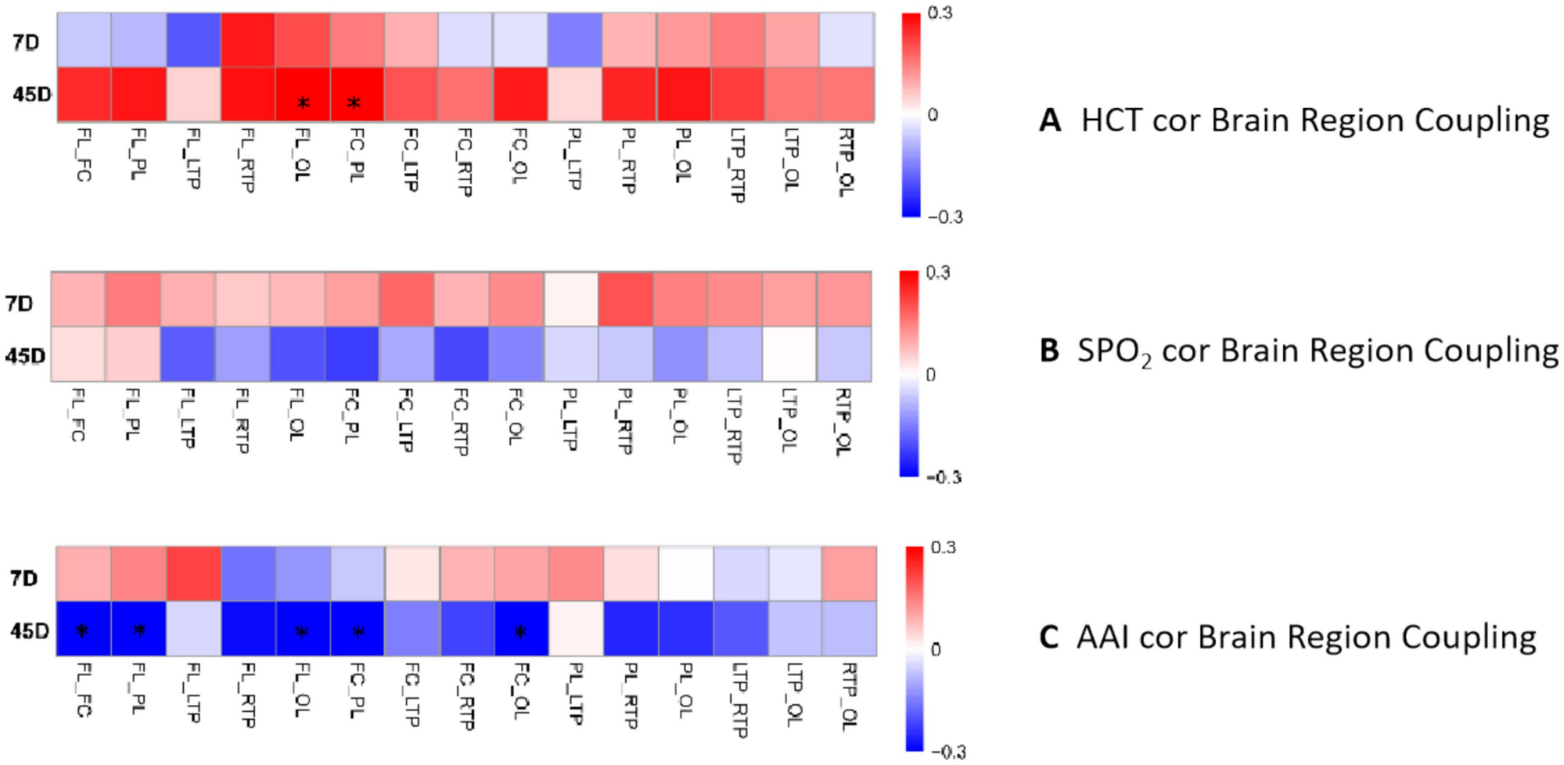
Partial correlation analysis between HCT, SpO₂, and AAI with coupled MSE between brain regions during acute (7D) and chronic (45D) exposure periods (covariates: AAI, gender, BMI at baseline). **(A)** HCT, **(B)** SpO₂, **(C)** AAI. During the chronic exposure period, AAI was significantly and negatively correlated with coupled MSE in the FL-FC, FL-PL, FL-OL, FC-PL, and FC-OL brain regions. *Represents *p* < 0.05.

In order to investigate the relationship between changes in physiological indicators and brain region coupling, we analyzed the correlations between SpO₂, HCT, AAI and brain region coupling during the acute and chronic exposure periods, respectively. It was found that AAI showed a significant negative correlation with MSE coupling of FL-FC, FL-PL, FL-OL, FC-PL, FC-OL brain regions during the chronic exposure period; whereas, we did not observe a direct significant correlation between SpO₂, HCT, and AAI and any brain region coupling during the acute exposure period (see Figure 7). Further observation revealed that AAI significantly decreased in the chronic exposure period compared to the acute exposure period. This suggests that the decrease in the physiological index AAI during the chronic exposure period directly affects the increase in the coupling between the FL-FC, FL-PL, FL-OL, FC-PL, and FC-OL brain regions. This shows that the increase in coupling between these brain regions during the chronic exposure period is closely related to the changes in AAI.

## 4 Discussion

In the present study, we attempted to elucidate the trends of brain resting-state signals in individuals from the acute acclimatization period to the chronic acclimatization period of high altitude exposure. We found that the MSE of six brain regions, frontal, central frontal, parietal, left temporoparietal, right temporoparietal and occipital, was significantly higher during the acute acclimatization period (within 7 days of Tibet entry) than during the chronic acclimatization period (45 days of Tibet entry), whereas the coupling between brain regions was significantly higher during the chronic acclimatization period than during the acute acclimatization period (frontal–parietal, frontal-occipital, central frontal–parietal, and central frontal-occipital). Meanwhile, our results showed that blood oxygen saturation decreased significantly after entering high altitude, and SpO₂ recovered slowly during the chronic acclimatization period with the prolongation of exposure time to high altitude hypoxia. HCT increased significantly from the acute acclimatization period until the chronic acclimatization period when it still remained at a high level, and at the same time, due to the fact that the increase in HCT was higher than that of SpO₂, the acute acclimatization period to the chronic acclimatization period, the AAI index showed a decreasing trend, but the decrease was slowed down in terms of magnitude. In order to further explore the relationship between individual adaptation and MSE and brain area coupling, we correlated SpO₂, HCT and AAI with MSE and brain area coupling during the acute adaptation period and chronic adaptation period, respectively, and found that, during the acute adaptation period, SpO₂ was negatively correlated with MSE at finer scales (1–6), HCT was negatively correlated with MSE at coarser scales (6–15), and HCT was negatively correlated with MSE at coarser scales (6–15), while AAI was positively correlated with MSE at the occipital lobe at the mesoscale (7–11), and there was no significant correlation between the three physiological indices and the coupling of brain regions. In contrast, during the chronic adaptation period, AAI was significantly negatively correlated with the coupling of FL-FC, FL-PL, FL-OL, FC-PL, and FC-OL brain regions, respectively, and there was no significant correlation between the three physiological indices and the MSE of each brain region.

SpO₂ is the percentage of hemoglobin (Hb) bound to oxygen in the blood, which is an important physiological parameter reflecting blood oxygenation (Zhang et al., 2004), a decrease in SpO₂ implies a decrease in the percentage of hemoglobin bound to oxygen in the blood, i.e., an insufficient supply of oxygen to the body tissues, after entering an acute low-pressure hypoxic environment, an individual’s SpO₂ decreases, but the body can increase its oxygen delivery through enhanced respiration, increased pulmonary ventilation and other compensatory body responses to increase oxygen delivery (Zhang et al., 2009), whereas elevated HCT is an adaptation to chronic hypoxia that can be initiated acutely by sympathetically mediated splanchnic contraction (Mayfield et al., 1994). In our study, we found that after entering the chronic acclimatization period, SpO₂ slowly rebounded, while, at the same time, HCT continued to increase and the decrease in the AAI index slowed down significantly, suggesting that after 45 days of exposure to high altitude hypoxia, individuals began to show adaptation to the hypoxic environment.

During the acute adaptation period, the MSE of brain regions was significantly higher than that in the chronic adaptation period, whereas inter-regional coupling showed the opposite pattern—significantly higher during the chronic period. These changes suggest that as adaptation progresses from acute to chronic, regional neural activity and complexity decrease, energy is temporally and spatially redistributed, and inter-regional coupling strengthens. We propose that this represents an adaptive response to prolonged hypoxia.

Spontaneous neural activity is a major driver of the brain’s high energy cost. Although the adult brain accounts for only 2% of body weight, it consumes 20% of the body’s energy budget (Raichle, 2015). Under chronic hypoxic conditions, the brain not only enhances oxygen delivery by remodeling capillaries and promoting angiogenesis (Boero et al., 1999), but also likely reduces spontaneous activity to lower energy demand, while simultaneously strengthening inter-regional coupling to preserve normal brain function. Previous studies using an extended calibrated BOLD method have demonstrated that hypoxic conditions with 12% O_2_ significantly reduced the amplitude of stimulus-evoked cerebral metabolic rate of oxygen (CMRO_2_) responses (Zhang et al., 2020), indicating that transient hypoxia alters cerebral oxygen metabolism. According to Fick’s principle, changes in OEF are related to changes in CBF, CMRO_2_, and arterial oxygen content. Although regional cerebral blood flow (CBF) shows no significant change under hypoxic conditions, the observed reduction in OEF suggests a decrease in regional CMRO_2_ under hypoxia (Yin et al., 2021). We believe that this decrease in complexity of activity but increase in coupling between brain regions is a functional compensatory mechanism for hypoxic environment.

This view is supported by existing studies. In a study of 16 Tibetan Plateau migrants, researchers used resting-state functional magnetic resonance imaging (fMRI) and diffusion tensor imaging (DTI) methods to demonstrate that long-term highland hypoxia habituation can alter functional and structural connectivity between the hemispheres of the human brain, and at the same time, they found coupled modifications of bilateral visual cortex, which suggests that functional connectivity between brain regions can be altered by long-term hypoxia habituation. This suggests that enhanced functional connectivity between brain compartments is one of the important neural compensatory mechanisms for visual dysfunction in high-altitude migrants (Chen et al., 2016), and another high-altitude hypoxia exposure tracking study on 23 college students also found enhanced functional connectivity in the bilateral lingual gyrus within the dorsal visual flow pathway, and this increase in dorsal flow functional connectivity may compensate for retinal photoreceptor cell function, thereby maintaining normal visual function (Zhang et al., 2024).

Brain adaptations are also important for organismal adaptation to hypoxic environments. A study of mild traumatic brain injury (mTBI) identified two compensatory patterns of altered brain functional connectivity: (1) hyperconnectivity between the posterior cingulate gyrus (PCC) and brain association areas and (2) an increase in frontal-occipital functional connectivity, demonstrating that the brain compensates by increasing functional connectivity at the connectome scale after injury (Iraji et al., 2016). The second pattern (frontal-occipital coupling) was confirmed in this study, while the first pattern (PCC hyperconnectivity) warrants further investigation using source localization techniques.

It has been demonstrated that multiple regions of the default network have increased functional connectivity to the PCC in patients with chronic altitude sickness, suggesting that alterations in multiple brain regions of the default network in the brain of patients with chronic altitude sickness in a long-term chronic hypoxic environment are mainly a compensatory mechanism (Kong et al., 2016). The default mode network (DMN) is a widely distributed group of brain regions in the parietal, temporal, and frontal cortices (Smallwood et al., 2021), and has been associated with functions such as emotion processing, self-referential mental activity, and recollection of prior experiences. In the present study, we found that during chronic adaptation, coupling between brain regions was enhanced mainly between four brain regions: frontal (FL), frontal-central (FC), parietal (PL) and occipital (OL), where the parietal and frontal cortices are involved in the composition of the default mode network, and the alterations of these brain regions as a part of the compensatory mechanism are consistent with the compensatory model (1), and at the same time, we found that during the chronic adaptation period, the frontal–parietal and frontal-occipital coupling between brain regions is enhanced during chronic adaptation, which is consistent with the compensatory model (2). But the ROIs in this study were defined based on electrode coordinates rather than cortical anatomical templates. Future studies incorporating source imaging could further validate the relationship between our findings and the DMN.

Both modes of compensation aim to maintain homeostasis in the brain. The free energy principle of adaptive systems (i.e., organisms, such as the brain) holds that any self-organizing system in equilibrium with its environment must minimize its free energy and thus resist the natural tendency toward disorder/entropy, i.e., to ensure that homeostasis is achieved within the system, i.e., in the face of changing environments, the brain tends to maintain their state and form, operating on the verge of a delicate equilibrium and avoiding a disordered state (Tozzi et al., 2016), and an increase in MSE may indicate that the brain networks have a greater tendency to transition or explore between states, and thus have a greater tendency to process information (Wang et al., 2018), so that during chronic adaptation, prolonged hypoxia results in the brain’s various brain regions to complexity appears to decrease, the transition states of the brain network become smaller, and more of this energy is used to strengthen the coupling between brain regions, which is a manifestation of the brain’s efforts to achieve a dynamic equilibrium between the organism and the environment.

Meanwhile, the present study also found that during the acute adaptation period, SpO₂ and HCT can affect the MSE values of brain regions at different scales, with SpO₂ showing a negative correlation with MSE at finer scales (1–6) and HCT showing a negative correlation with MSE at coarser scales (6–15). Fine time scales reflect interconnectivity between local neural populations, whereas coarse time scales reflect long-range interactions between distributed neural populations (Vakorin et al., 2011; McIntosh et al., 2014), implying that, during acute acclimatization, a decrease in SpO₂ leads to an increase in local neural population interconnections to rise, generating higher complexity at finer scales and the emergence of more localized information processing (Courtiol et al., 2016), i.e., greater modular independence across brain regions; whereas a rise in HCT leads to a reduction in long-range interactions between distributed neural populations, i.e., a reduction in the integration of functional brain modules, which coincides with the emergence of higher brain regions during acute acclimatization of MSE coincides with this.

In contrast, during the chronic acclimatization period, AAI showed a significant negative correlation with the coupling of the MSE in the FL-FC, FL-PL, FL-OL, FC-PL, and FC-OL brain regions, i.e., the coupling between the brain regions increased as AAI decreased. As mentioned earlier, the increase in coupling between brain regions may be regarded as a mechanism of brain adaptation to the environment during chronic hypoxic acclimatization, and AAI, as an index for judging the individual’s plateau acclimatization habituation status, has the potential to be a valid predictor of coupling between brain regions, but as the decline in individual AAI levels during the chronic acclimatization period (45 days) selected for the present study, although a slowing down trend was observed, it still did not reach the final stable value, this hypothesis still needs to be proved by more in-depth studies in the future.

## 5 Limitations

Although the present study has some findings, it also has some shortcomings. Due to practical conditions, we were unable to collect resting-state EEG data from participants during the plains period in this study, and thus were unable to compare the resting-state EEG activity during the acute acclimatization period and chronic acclimatization period with that during the plains period, and we will make up for this part of the shortcomings in the future, and in the meantime, we will further follow up the participants to explore the complexity of brain and brain region coupling characteristics of high-altitude transplants following long-term exposure to high-altitude hypoxic environments and coupling of brain regions, and add high-altitude world-dwellers as a control group to further explore whether these brain activity characteristics have stable group qualities, and finally, we will also try to add participants to the experiments after reoxygenation to explore whether the brain activities found in this study are reversible.

## 6 Conclusion

The current literature lacks direct use of MSE to explore the effects of high-altitude hypoxic environments on brain complexity and functional connectivity, and our findings add to this picture, while also suggesting perspectives for future research. In addition to traditional SpO₂ and HCT metrics, we demonstrated the role of AAI metrics in assessing individual plateau acclimatization habituation and predicting functional brain connectivity. We found that a decrease in SpO₂ leads to a rise in local neural activity and a rise in HCT leads to a decrease in long-range interactions between neural populations, resulting in a higher complexity of activity in brain regions during acute acclimatization, whereas with an increase in the duration of exposure to high-altitude hypoxia, the complexity of activity in each brain region decreases and there is a spatial and temporal redistribution of energy, which tends to strengthen coupling between the various brain regions. We believe that this is a kind of compensation for the brain’s adaptation to the hypoxic environment, but this needs to be proved by more in-depth studies in the future.

## Data Availability

The raw data supporting the conclusions of this article will be made available by the authors, without undue reservation.

## Footnotes

1
https://www.ant-neuro.com
