# Supplementary material for: Relationship between spontaneous EEG oscillations at 7 and 45 days of acute plateau exposure and the plateau acclimatization index
Source: Front Neurosci. 2026 Jun 18;20:1830546. doi: 10.3389/fnins.2026.1830546 (PMC13323323; doi:10.3389/fnins.2026.1830546)
Supplement: Supplementary file 1 [file Supplementary_File_1.docx]

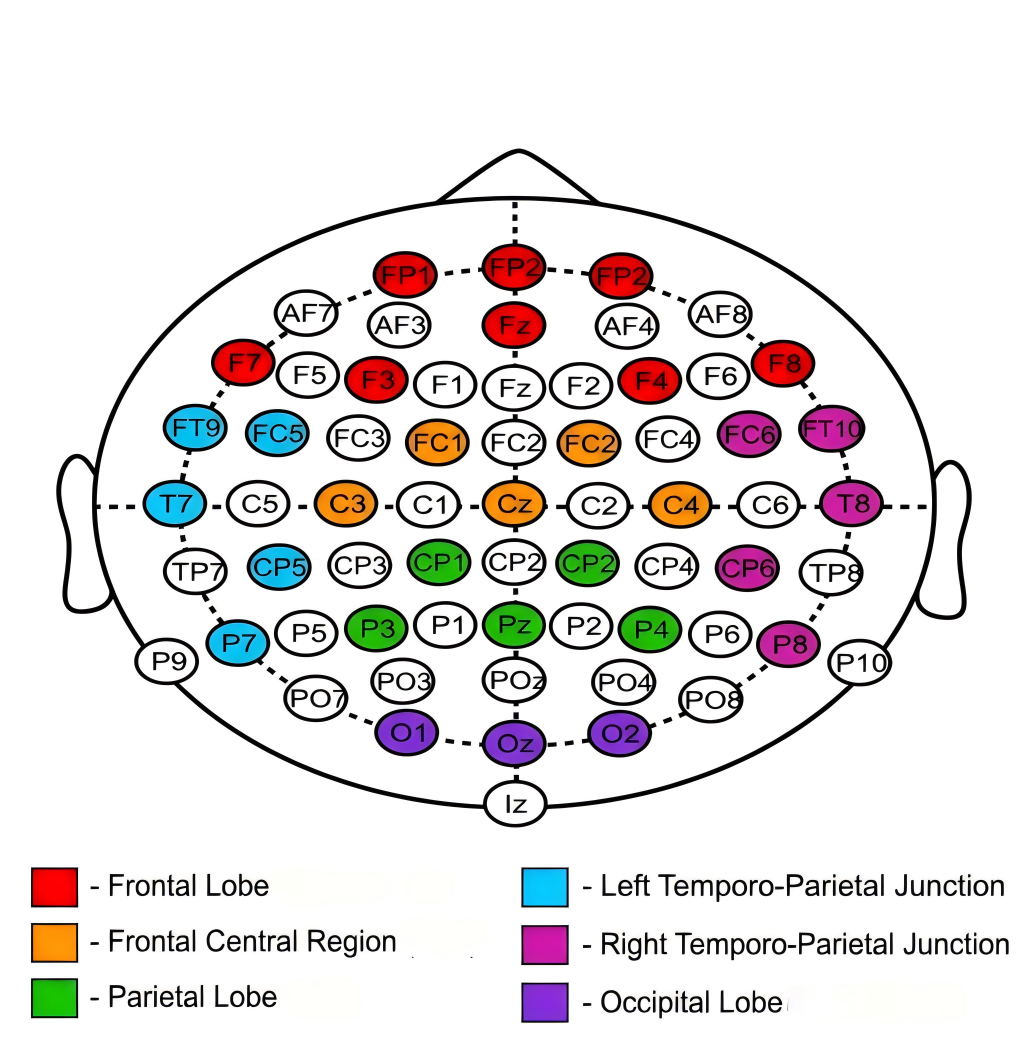
**Supplementary Materials**

**SUPPLEMENTARY FIGURE 1** Schematic diagram of the electrode layout highlighting the six regions of interest (ROI). (A) Top view of the full scalp electrode distribution; (B) Illustration of the six ROIs: frontal lobe, frontal central region, parietal lobe, left temporo-parietal junction, right temporo-parietal junction, and occipital lobe. The specific electrodes within each ROI are color-coded as indicated in the legend.
